# Surgical Management of Thoracic Arachnoid Cyst Causing Spinal Cord Compression: A Case Report

**DOI:** 10.7759/cureus.96378

**Published:** 2025-11-08

**Authors:** Hector R Peña-Popo, Diego Molina-Botello, Edgar F Higuera-Gonzalez, Andrea García-Bitar, Felix Dominguez-Cortinas

**Affiliations:** 1 Neurological Surgery, Hospital de Especialidades, Centro Médico Nacional Siglo XXI, Instituto Mexicano del Seguro Social, Mexico City, MEX

**Keywords:** arachnoid cyst, spinal arachnoid cyst, spinal cord compression, spinal magnetic resonance imaging, thoracic spine surgeries

## Abstract

Spinal arachnoid cysts are rare intradural spinal canal collections of cerebrospinal fluid enclosed by an arachnoid membrane, often associated with congenital or acquired dural defects, most frequently located in the thoracic spine, although they may also occur at lumbar or cervical levels. Symptomatic arachnoid cysts typically cause progressive neurological deficits secondary to spinal cord or nerve root compression. We report the case of a 29-year-old woman presenting with a 1-year history of progressive low back pain, lower limb weakness, and persistent pain unresponsive to conservative management. Magnetic resonance imaging (MRI) demonstrated an extramedullary, extradural cystic lesion extending from T9 to L2, causing significant spinal cord displacement, consistent with an arachnoid cyst. The patient underwent microsurgical decompression consisting of a laminectomy and cyst fenestration under microscopic visualization, resulting in symptomatic relief. Postoperative imaging confirmed resolution of the spinal cord compression. This case underscores the necessity of timely diagnosis and surgical intervention in symptomatic spinal arachnoid cysts to prevent permanent neurological sequelae. MRI remains essential for accurate diagnosis, and early surgical treatment offers a favorable prognosis with low recurrence risk.

## Introduction

Spinal arachnoid cysts (SACs) are rare central nervous system lesions located within the spinal canal and filled with cerebrospinal fluid (CSF). These cysts, delineated by the arachnoid membrane, arise from dural abnormalities that allow CSF to accumulate. Although they may be idiopathic in origin, secondary causes include trauma, infections, prior surgical interventions, or inflammatory processes. Anatomically, these cysts are most commonly found in the thoracic spine, followed by the lumbar region, and less frequently in the cervical spine [1-3].

We describe the case of a young woman who presented with chronically evolving neurological symptoms. The patient reported persistent low back pain associated with lower limb weakness. Due to the failure of conservative management, magnetic resonance imaging (MRI) was performed, revealing a cystic lesion located extradurally between T9 and L2. The lesion was interpreted as an arachnoid cyst causing significant spinal cord displacement, which prompted surgical intervention.

The patient underwent microsurgical decompression consisting of laminectomy and cyst fenestration under operating microscope guidance for a thoracolumbar arachnoid cyst extending from T9 to L2. The clinical course was favorable, with progressive improvement of symptoms and no evidence of recurrence during initial follow-up. This case highlights the importance of early recognition of symptomatic spinal arachnoid cysts (SACs) and reinforces the role of surgical intervention as the primary therapeutic strategy to prevent irreversible neurological deterioration [1,3,4].

## Case presentation

We present the case of a 29-year-old female with a 1-year history of acute, constant low back pain, rated as 5/10 on the visual analog scale (VAS). The pain worsened with walking and improved partially with analgesics. Additionally, the patient reported a sensation of heaviness in the right lower limb, with progressive weakness, cold sensation, and paresthesias. Initially, she underwent physical therapy and rehabilitation, which led to complete symptom resolution within one month. However, six months later, symptoms recurred without an identifiable trigger, this time with bilateral lower limb involvement, eventually requiring the use of a walking stick for ambulation. A new course of analgesics and rehabilitation was initiated, but without significant clinical improvement.

Neurological examination revealed decreased muscle strength in the left lower limb (4/5) and in the right lower limb (5/5) according to the Daniels scale. Hypoesthesia was noted in the left lower limb, with a sensory level at L3, but sphincter control remained intact.

**Figure 1:**
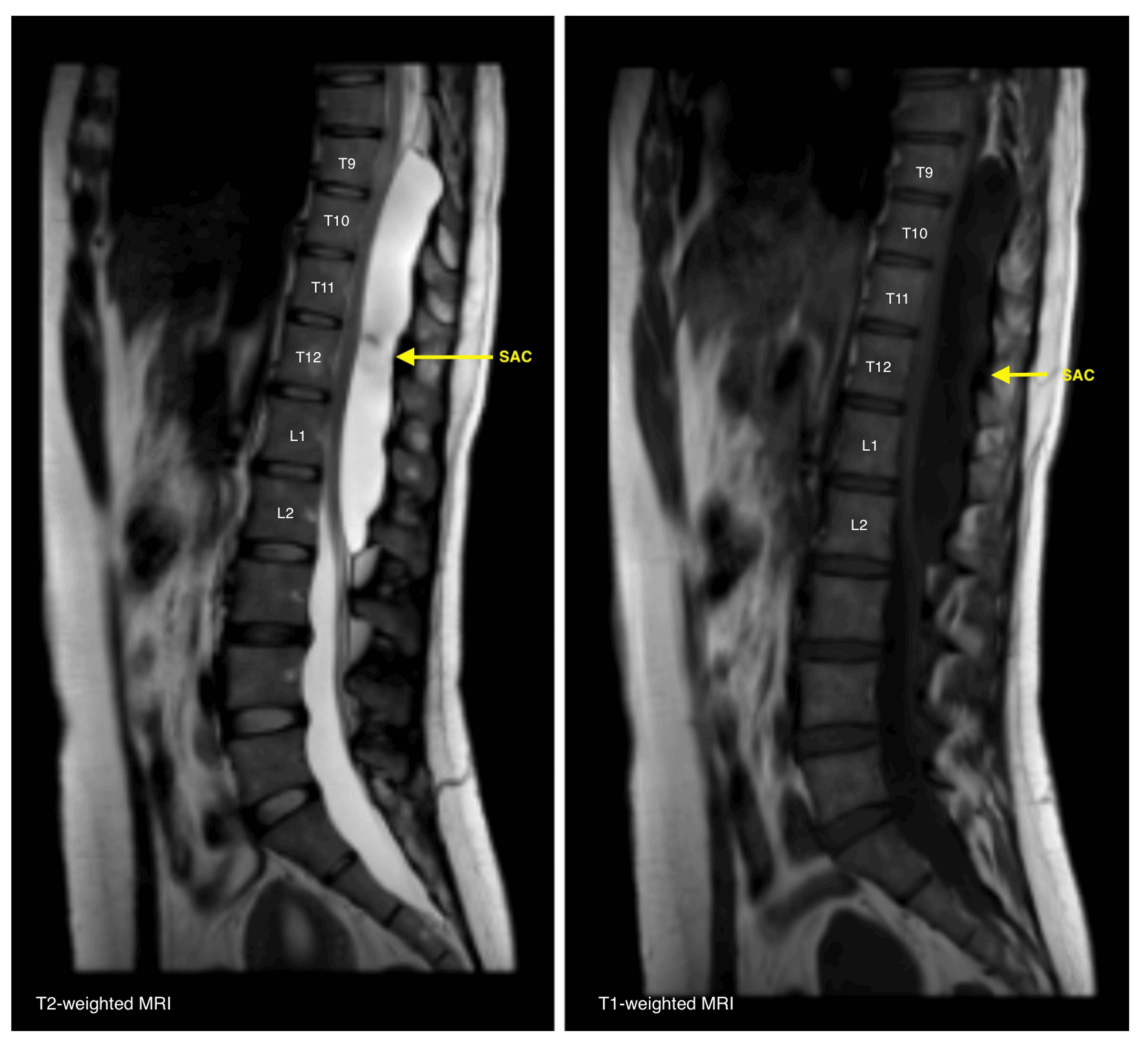
Preoperative sagittal T2-weighted magnetic resonance imaging (MRI) of the thoracolumbar spine demonstrating an extramedullary, extradural cystic lesion extending from T9 to L2 The lesion appears isointense to cerebrospinal fluid and causes anterior displacement of the spinal cord.

**Figure 2:**
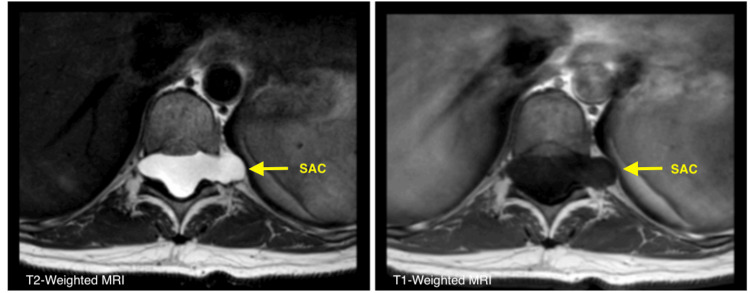
Preoperative axial T2-weighted MRI showing bilateral extension of the cystic lesion into the neural foramina, with greater involvement on the left side The lesion is isointense to cerebrospinal fluid and compresses adjacent neural structures.

Preoperative MRI of the lumbosacral spine demonstrated an apparently extradural, extramedullary lesion that was isointense to cerebrospinal fluid on both T1- and T2-weighted sequences, with no enhancement after gadolinium administration. The lesion extended from T9 to L2, measuring 148 mm in craniocaudal length and 20 mm anteroposteriorly, and caused significant anterior displacement of the spinal cord (Figure 1). Bilateral foraminal extension was observed, more prominent on the left side (Figure 2).

The patient underwent posterior thoracolumbar decompression through a T11 laminectomy and adjacent T10-T12 hemilaminectomies, with microscopic cyst fenestration and creation of a rostrocaudal communication within the cystic cavity.

**Figure 3:**
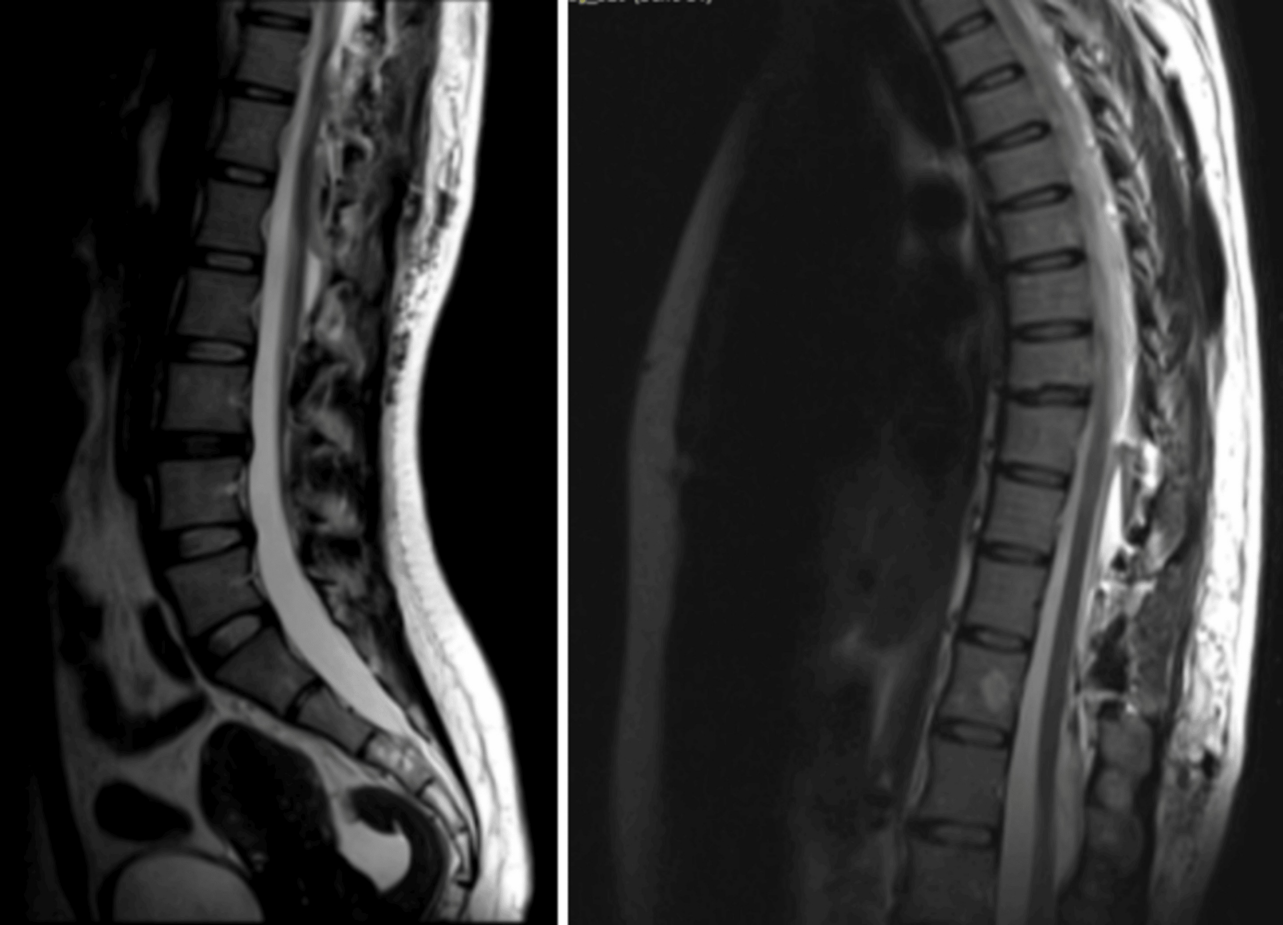
Postoperative sagittal T2-weighted MRI of the thoracolumbar spine showing resolution of the previously visualized extradural cyst and decompression of the spinal cord Postsurgical changes are evident at the T10–T12 levels.

**Figure 4:**
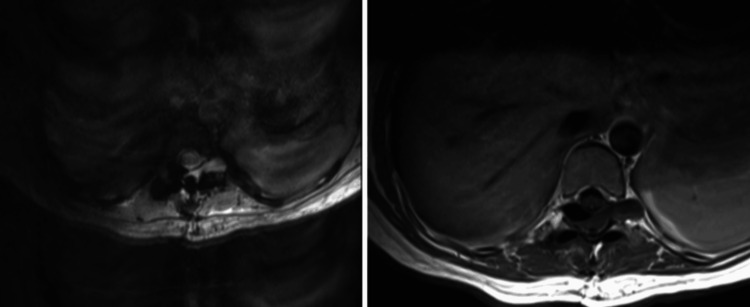
Postoperative axial T2-weighted MRI confirming absence of the cystic lesion and restoration of the spinal cord’s normal position, with no residual compression

Postoperative magnetic resonance imaging (MRI) demonstrated post-surgical changes, with resection of the lesion and adequate decompression of the spinal cord (Figures 3, 4).

## Discussion

SACs are uncommon lesions, accounting for approximately 1%-3% of intradural spinal masses. They are defined as loculated accumulations of CSF within the spinal canal, partially or completely enclosed by the arachnoid membrane, with histologic features indistinguishable from normal arachnoid tissue. Their development is typically associated with dural defects, either congenital or acquired, which create a one-way communication with the subarachnoid space. This mechanism, often described as a “ball-valve” effect, facilitates the progressive accumulation of CSF. SACs are most commonly located in the intradural extramedullary compartment, surrounding the spinal cord or nerve roots [1-3,5-8].

From an epidemiological standpoint, most series agree that SACs occur most frequently between the fourth and sixth decades of life [3], with a mean age at diagnosis ranging from 40 to 58 years [2,4,9]. Sex distribution is inconsistent across studies: while some report a higher incidence in females, up to a 3:1 ratio [2], others describe a slight male predominance [5,9,10]. In 1 series of 10 cases, for instance, the mean age was 40 years (range: 3-59), with a female-to-male ratio of 1.5:1 [4].

The thoracic region is the most frequently affected site, reported in 65%-77% of cases, followed by the lumbar spine (13-25%) and the cervical spine (3-10%). However, cases involving multiple contiguous segments, such as cervicothoracolumbar cysts, have also been described [2,3,10].

From an anatomical standpoint, SACs are classified based on their relationship to the dural sac into three main types: extradural communicating cysts (EDACs), extradural non-communicating cysts (IDACs), and intramedullary arachnoid cysts (IMACs). EDACs are the most common subtype, accounting for approximately 90% of cases, whereas intramedullary cysts are exceptionally rare.

Acquired causes include spinal trauma, infections such as meningitis, subarachnoid hemorrhage, prior surgeries, and invasive procedures like myelography or lumbar puncture [2,3]. These insults may produce localized damage to the arachnoid membrane, promoting cerebrospinal fluid accumulation through a ball-valve mechanism [1,3]. Additionally, connective tissue and inflammatory disorders, such as Marfan syndrome or multiple sclerosis, have been proposed as potential predisposing factors [10]. In some cases, SACs may arise spontaneously or in association with idiopathic subarachnoid fistulas, complicating etiological classification [3]. Although many patients present without a relevant clinical history, the possibility of a secondary cause should always be considered, particularly in those with a history of spinal interventions, trauma, or infectious processes [9,11].

The most widely used classification in clinical practice is that proposed by Nabors and colleagues in 1988. This system categorizes SACs into three types: Type I, extradural cysts without inclusion of nerve roots, such as sacral meningoceles; Type II, extradural cysts that contain nerve roots, commonly referred to as Tarlov cysts; and Type III, intradural cysts. This classification provides a structured framework for both diagnostic evaluation and therapeutic decision-making [1].

The clinical utility of this classification has been supported by observational data. In a series published by Fam et al., half of the identified SACs were intradural (Type III), while 27% were extradural, emphasizing the importance of considering both types in the diagnostic approach [2]. Similarly, Eroglu et al. reported that 54% of cases in their cohort were intradural and 38% extradural, highlighting that although extradural cysts are more prevalent overall, intradural cysts present a significant surgical challenge due to their proximity to the spinal cord [1].

The clinical presentation of SACs is highly variable and depends on factors such as the size of the cyst, its location within the spinal canal, and the degree of compression exerted on neural structures. In many cases, these lesions may be incidental findings with no evident clinical manifestations [4,7]. However, when symptomatic, SACs typically present with an insidious and progressively worsening clinical course [1,6,9].

Axial or radicular pain is the most frequently reported symptom, present in the vast majority of patients, followed by sensory disturbances, such as paresthesias or hypoesthesia, and motor deficits that may involve one or more extremities. In some series, motor and sensory impairments have been observed in 45% to 60% of patients [1,2,9,11]. Gait disturbances are also common and have been reported in over half of the cases involving spinal cord compression. In contrast, sphincter dysfunction is less prevalent but may occur, particularly when the cyst is located at sacral levels or compresses the cauda equina [1,2,4,11].

The location of the cyst along the spinal axis typically determines the predominant clinical pattern [3,7,10]. In addition, symptoms often fluctuate or worsen with maneuvers that increase intrathecal pressure, such as the Valsalva maneuver or postural changes [6,7,10]. In cases of persistent CSF leakage, intracranial hypotension may develop, clinically manifesting as orthostatic headache [3,6].

Diagnosis is based on the correlation between clinical findings and imaging studies. MRI is the diagnostic modality of choice, as it allows for the visualization of lesions with signal characteristics similar to CSF. These typically appear hypointense on T1-weighted sequences and hyperintense on T2-weighted sequences, often accompanied by signs of spinal cord compression. A characteristic finding in some cases is the angular displacement of the spinal cord, known as the “scalpel sign” [2,5,9,12].

Cine MRI can assess pulsatile cerebrospinal fluid (CSF) flow dynamics, while computed tomography myelography (CT myelography) is useful for identifying dural defects and fistulous tracts [1-3,7,9,10]. These tools are particularly valuable when conventional MRI does not provide sufficient information to establish a definitive diagnosis.

The differential diagnosis includes various conditions that may mimic SACs both clinically and radiologically. These include syringomyelia, perineural cysts (such as Tarlov cysts), intradural tumors (e.g., ependymomas, schwannomas), dermoid or epidermoid cysts, lipomas, and adhesive arachnoiditis. Less common entities, such as dural ectasia, spinal vascular malformations, and spontaneous spinal cord herniation, should also be considered [3,5,8,9].

In some cases, the definitive diagnosis is only confirmed intraoperatively through direct inspection of the cyst and verification of its communication with the subarachnoid space [3]. Therefore, a comprehensive diagnostic approach, integrating a detailed clinical history, advanced imaging studies, and, when indicated, surgical exploration, is essential to establish an accurate diagnosis and to rule out similar pathologies.

The most commonly performed procedure is complete cyst resection, which is considered the most effective option due to its low recurrence rate. However, in cases where the cyst’s anatomy precludes total resection, such as ventrally located lesions or those with dense adhesions, alternative techniques, such as marsupialization or fenestration, may be employed to create communication with the subarachnoid space and reduce pressure [2].

In selected cases, particularly with recurrent cysts or lesions that are difficult to access, additional procedures may be necessary, including ligation of the communicating tract with the subarachnoid space or placement of a cystoperitoneal shunt. For extradural cysts, complete excision combined with closure of the dural defect is preferred, whereas intradural cysts typically require a dural opening (durotomy) to facilitate resection or drainage. The choice of surgical approach--posterior, anterior, or combined--should be tailored to the cyst’s location, extent, and anatomical characteristics [2].

When treatment is performed in a timely manner, prior to the establishment of permanent neurological damage, outcomes are generally favorable. Improvement in pain and muscle weakness is commonly observed after surgery. However, some patients may continue to experience residual sensory disturbances or sphincter dysfunction, particularly when there is pre-existing damage to neural structures. In long-term follow-up studies, recurrence has been reported as rare when complete cyst resection is achieved and dural closure is adequately secured [6].

Reviewed studies report generalized motor improvement in all surgically treated patients, while sensory symptoms tend to resolve partially. In cases of recurrence, surgical reintervention or cyst content diversion (e.g., cystoperitoneal shunting) has been shown to yield favorable clinical outcomes [9].

## Conclusions

SACs are rare, potentially reversible causes of spinal cord compression. Although their clinical presentation is often insidious and nonspecific, progressive neurological decline warrants timely diagnosis and surgical management. Magnetic resonance imaging remains the cornerstone for detection and characterization, enabling differentiation from other cystic or neoplastic lesions. Microsurgical decompression through laminectomy and cyst fenestration under operative microscopy provides effective and durable symptom relief by restoring normal cerebrospinal fluid dynamics. Early surgical intervention, before the onset of irreversible neurological injury, offers the best prognosis and minimizes recurrence. SACs are lesions that may or may not be symptomatic, and for clinical and imaging diagnosis, a high degree of diagnostic suspicion must be maintained. When defining the surgical plan, individualize cases. In our case, we opted for the aforementioned surgery, with a very favorable outcome.

The present case underscores the importance of clinical suspicion and prompt neurosurgical evaluation in patients presenting with progressive paraparesis and imaging findings consistent with spinal arachnoid cysts.
